# Evaluation of the Efficiency of the Atraumatic Endotracheal Tube in the Pulmonary-Gas Exchange: an Experimental Study

**DOI:** 10.5935/1678-9741.20150089

**Published:** 2015

**Authors:** Raíssa Quaiatti Antonelli, Marcos Mello Moreira, Luiz Claudio Martins, Maíra Soliani Del Negro, Tiago Antonio Baldasso, Alfio José Tincani

**Affiliations:** 1 Universidade Estadual de Campinas (Unicamp), Campinas, SP, Brazil

**Keywords:** Gases, Intubation, Intratracheal, Animal Experimentation, Oxygenation, Pulmonary gas Exchange, Capnography

## Abstract

**OBJECTIVE:**

Mechanical ventilation is frequently necessary, in which case the use of an endotracheal tube is mandatory. The tube has an inflatable balloon in its distal extremity, whose aim is, among other functions, an efficient arterialization. However, serious injuries in the place of contact of the balloon with the trachea can be frequent. Some studies point out that balloons with permanent pressure may reduce this complication. Nevertheless, air scape, expressed by the inspiratory (IV) and expiratory volume (EV) variation (Δ IV-EV), may occur, possibly leading to hypoxemia. Thus, the goal of this study was to verify the efficiency of a modified endotracheal tube on arterializations compared to the traditional endotracheal tube.

**METHODS:**

The modified endotracheal tube presents intermittent insufflation, with three drillings in the internal region of the cuff, allowing for insufflation in the inspiratory phase of the mechanical ventilation. Three animals were used for the control group, with a cuff pressure of 30 cmH_2_O, and seven pigs had the modified endotracheal tube. Each animal was kept under mechanical ventilation (FIO_2_=0.21) for 6 hours. Arterial and venous gases were measured every three hours (T_0_; T_3_; T_6_).

**RESULTS:**

The gases confirmed the lack of hypoxia between the Groups, with a difference in the ΔIV-EV at T0 (*P*=0.0486).

**CONCLUSIONS:**

In this study, the lack of hypoxia showed the efficiency of the modified endotracheal tube. However, new studies are necessary, particularly in diseased lungs, in order to evaluate the real efficiency of the mentioned device on the pulmonary gas exchange.

**Table:** 

**Abbreviations, acronyms & symbols**	
ARDS	= Acute respiratory distress syndrome
ETT	= Endotracheal tube
EV	= Expiratory volume
IMV	= Invasive mechanical ventilation
IV	= Inspiratory volume
METT	= Modified endotracheal tube
PEEP	= Positive end-expiratory pressure
TETT	= Traditional endotracheal tube

## INTRODUCTION

Mechanical assistance (invasive and non-invasive) to pulmonary ventilation is frequently necessary for the successful treatment of acute lung insufficiency and thus it is considered an important measure capable of saving the life of patients under critical conditions^[1,2]^. The traditional endotracheal tube (TETT) is largely employed in the medical area, particularly on patients who demand invasive mechanical ventilation (IMV).

The TETT holds terminal balloons (cuffs) which, when inflated, occlude the space between the tube and the tracheal wall, thus not allowing the air to scape. The balloons are also capable of preventing the entrance of solid residues and fluids, such as colonized secretions of the oropharynx and gastric content, which could lead to a series of complications such as respiratory infections and pneumonitis. Those same inflatable balloons can cause serious lesions in the contact area with the trachea, and maybe worsen a specific treatment (IMV)^[2]^.

Current *in vitro* studies show that lower entrance of fluid secretions may occur, as shown in a study using dye (methylene blue) inserted in the oropharynx of animals (pigs) with TETT^[2-4]^.

Other studies show the complications which may occur in the tracheal wall due to the continuous use of the TETT, especially stenosis^[5-10]^. Two main mechanisms that contribute to stenosis are the downward movement of the tubes^[6,11]^, many times simply because of the inspiratory and expiratory cycle, and the damages caused to the mucosa from the pressure applied by the balloon^[2,5,11]^.

However, certainly the main factor causing lesions in the tracheal wall is the excessive pressure of the cuff, even in cases of low pressure^[2,5,11]^.

Experimentally, it can be verified that the pressure of capillary perfusion in the mucosa of rabbits' tracheas ranges from 25 to 30 mmHg^[10]^. Therefore, the pressure of the balloons that overlaps these values, even if applied for a short period of time, is the most likely cause of the pre-disposition of the tracheal mucosa to circulatory diseases by vascular compression and, consequently, to necrosis by ischemia^[5-7,11]^.

As an effort to reduce the damages caused by the balloon of the endotracheal tubes (ETT), several changes in the ventilation method, new equipment for pressure control as well as alterations in the balloon's design were suggested^[6]^. A handful of practices have already been developed, such as regular disinflation, alternate insufflation of a double balloon, insufflation only doing respiration, and even careful insufflation until complete closure. Unfortunately, such practices have so far been inefficient regarding tracheal lesions^[2]^.

In the 1970's, Nordin et al.^[10]^ and Grillo et al.^[12]^ clearly established that the use of balloons with low pressure and high complacence reduced the incidence and the gravity of the lesions in the patients' tracheas^[2,5,7]^.

In the auto-inflatable and permanent model set up by Abouav & Finley^[5]^, increase and decrease of the cuff's pressure occurred, followed by periods of insufflation and disinflation, making it possible to automatically adjust the closure pressure to the intratracheal one in the insufflation peaks. Their data differed from what happens in the regular balloons, in which the pressure is continuous even after an initial inflation. Clinical implications of those results were the lack of massive aspiration of content of the oropharynx and the maintenance of reasonable concentration levels of gases in the arterial blood of observed patients. Such proposed model included long-term observation of 52 patients under IMV (self-inflatable model) in addition to 200 laboratorial observations showing lack of tracheal lesions. The study aimed to analyze the pressure of the balloon in the self-inflatable and in the traditional model, besides seeking to understand the histology of the tracheal mucosa (indicating tracheal necrosis by compression and ischemia). It showed promising results regarding a new model, however, without the proposition to analyze pulmonary gas exchange. Parallel to this clinical trial, in an experimental study conducted on dogs, when analyzing the trachea six weeks after tracheostomy, the authors noticed it was free from any ulcers.

After intense search in the literature, few were the studies that clarified the experimentations in animal models in regard to pulmonary gas exchange.

The current study presents capnographic and blood gas analysis for the evaluation of pulmonary gas exchange during ventilation with TETT. According to research on the databases, no study has aimed to evaluate the efficiency of pulmonary gas exchange at modified TETT.

Examples of assessment of pulmonary gas exchange (capnographic and blood gas analysis) are found in experimental^[13,14]^ and clinical trials^[15,16]^ of pulmonary thromboembolism, as well as in clinical^[17-19]^ and experimental^[20,21]^ studies on acute respiratory distress syndrome (ARDS) and bronchopleural fistula^[22,23]^.

Thus, it has been observed that until the present moment none of the proposed methods aiming to minimize IMV inherent complications was efficient^[5]^.

As mentioned above, studies point out that balloons with higher volume, low pressure, and intermittent internal pressure appear to indicate a better interface. Therefore, the purpose of the current study was to verify the efficiency of a proprietary modified endotracheal tube (METT), which was developed and studied in the sense of reducing possible lesions in the tracheal wall^[4]^. In this paper, the aim was to verify its efficiency regarding pulmonary gas exchange (arterialization), in which the capnographic, arterial and venous gases were used under IMV and, randomly, with a FiO_2=_0.21.

## METHODS

The study was approved by the Ethics Committee for Animal Experimentation of the Biology Institute of University of Campinas (Unicamp) under protocol number 2612-1.

Continuing the study initiated and developed by Servin et al.^[4]^, in this study, 10 pigs from the Large White breed (30 kg) were used. Out of the 10, only three were allocated to the control group, which received the TETT with 30 cmH_2_O of cuff pressure; the remaining seven animals used the METT (cuff pressure was not measured because these tubes do not have a pilot tube). All tubes had internal measure of 6 mm (# 6.0 mm). The DX 3010^®^ Dixtal mechanical ventilator was employed, cycled to volume, with tidal volume from 10 to 15 ml/kg so that the end-tidal CO_2_ (EtCO_2_) remained around 40 mmHg; the positive end-expiratory pressure (PEEP) was 5 cmH_2_O and the FiO_2_=0.21. These ventilating parameters were kept throughout the experiment.

Between the ETTs and the IMV circuit, flow and reading sensor of exhaled CO_2_ (Capnostat^®)^ from the respiratory profile monitor CO_2_MO PLUS DX-8100^®^ (Dixtal/Novametrix, São Paulo, Brazil) were used, which enabled the calculation of the difference between inspiratory and expiratory volumes (IV and EV, respectively).

**Fig. 1:**
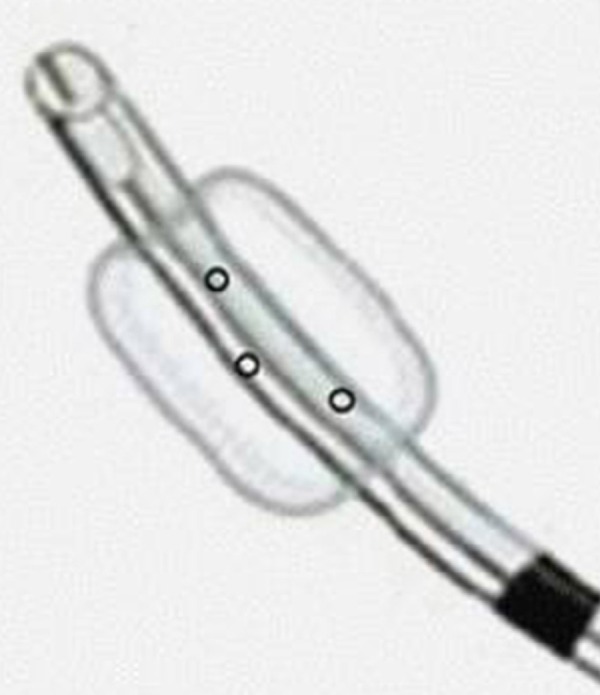
Schematic drawing of the modified endotracheal tube (METT).

**Fig. 2:**
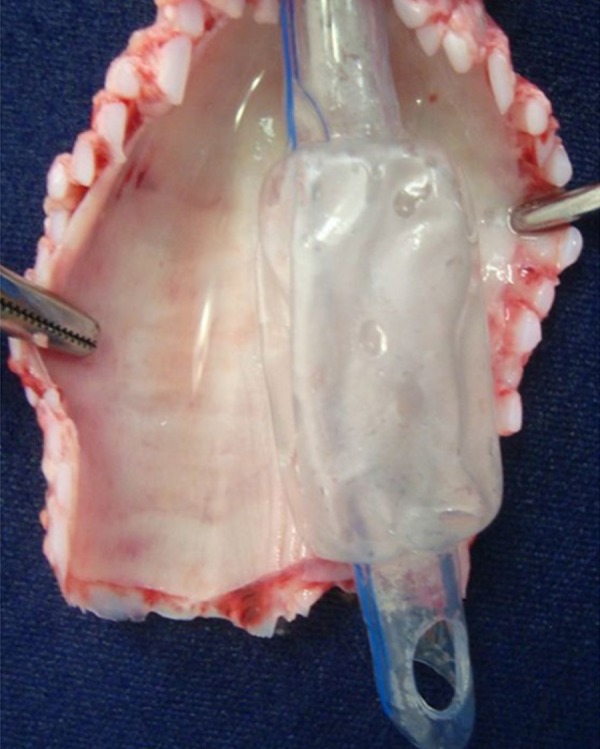
Modified endotracheal tube (METT) at trachea (*post mortem*).

**Fig. 3:**
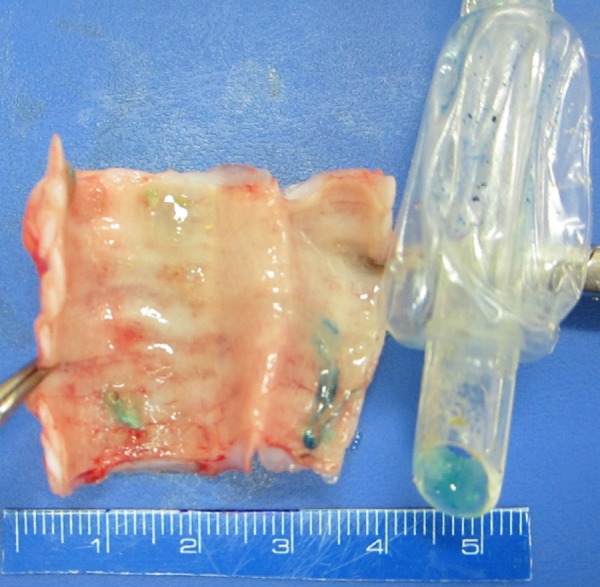
Traditional endotracheal tube (TETT) at trachea (*post mortem*).

Regarding the METT, it presented three drillings of 3 mm each in the region of the distal balloon (Figures 1 and 2). Insufflation and disinflation of the balloon occurs with the variation in pressure according to the respiratory cycle of the IMV. The Figure 3 shows TETT at trachea (*post mortem*).

**Fig. 4:**
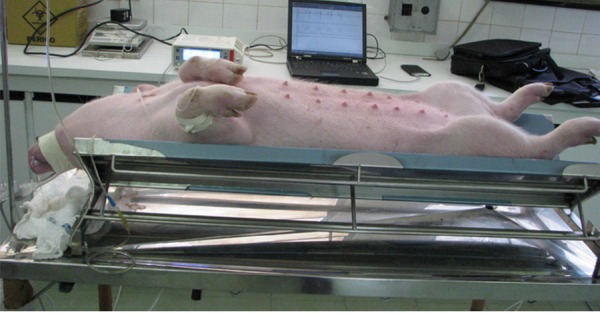
Overview procedure.

The animals were hemodynamically monitored with access to mean arterial pressure and heart rate (Figure 4).

Each animal remained anesthetized with sodium thiopental and pancuronium for a continuous period of six hours. Arterial and venous blood gases were collected every three hours, as follows: T_0_ (baseline), T_3_ (three hours after T_0_) and T_6_ (six hours after T_0_).

The data collection of the gases (both arterial and venous), and the respiratory and hemodynamic assessment were performed on the ten animals.

On completion of the experiment, the animals were sacrificed with thiopental overdose and potassium chloride injection.

Statistical analysis

The data were described through means, medians, standard deviation, or frequencies.

Comparison of the groups was performed each time through the Mann-Whitney nonparametric test (due to the n=3 in the control group), and between times in the experimental group through the paired Wilcoxon non-parametric test. A normality test was performed.

Level of significance was assumed at 5% and the software used for analysis was SAS version 9.2.

## RESULTS

Numerical analysis regarding the PaO_2_ data was not statistically significant for this variable when comparing the two groups (TETT and METT) and the times (T_0_, T_3_, T_6_).

There was a significant difference between the groups regarding HCO_3_ in the T_3_ instant (*P*<0.0486) and ΔIV-EV in T_0_ (*P*=0.0486) (Mann-Whitney test).

There were statistical differences between T_0_ and T_3_ in relation to venous pH (*P*=0.0469) and heart rate (*P*=0.0313) (Wilcoxon test), and between the times T_3_ and T_6_ regarding oxygen saturation of venous blood (Sat.v O_2_) (*P*=0.0156) for the TETT (paired Wilcoxon test).

The results obtained in this study were summarized on Tables 1 to 4.

**Table 1:** Blood gas data of the METT (n=7) and the TETT (n=3) Groups at T_0_

T_0_												
	pH	PaO_2_	PaCO_2_	BE	hco_3_	Sat.O_2_	pH v	PvO_2_	PvCO_2_	BE v	HCO_3_ v	Sat.v O_2_
METT	7.48±0.06	83.3±3.54	41.3+0.1	6.3±4.2	29.5±4.3	93.9+0.8	7.39+0.08	43.0+11.1	56.7+5.9	6.9+3.7	32.0+3.1	41.7+23.7
TETT	7.47±0.04	104.1±9.0	37.7+1.2	3.3±2.4	26.2+2.0	97.3+1.1	7.38+0.05	44.9+7.5	47.8+6.8	3.5+3.4	27.8+3.1	57.7+16.8

METT=modified endotracheal tube; TETT=traditional endotracheal tube; PaO_2_=partial pressure of oxygen in arterial blood; PaCO_2_=partial pressure of carbon dioxide in arterial blood; BE=base difference in the arterial blood; HCO_3_=bicarbonate in arterial blood; Sat.O_2_=hemoglobin saturation in arterial blood; PvO_2_=partial pressure of oxygen in venous blood; PvCO_2_=partial pressure of carbon dioxide in venous blood; BE v=base difference in venous blood; HCO_3_ v=bicarbonate in venous blood; Sat.v O_2_=hemoglobin saturation in venous blood.

Results expressed as mean ± standard deviation.

**Table 4:** Respiratory and hemodynamic data of the METT (n=7) and the TETT (n=3) Groups.

Group METT (n=7)				Group TETT (n=3)		
	Δ IV-EV	MAP	HR	Δ IV-EV	MAP	HR
^T^0	54+42	89+16	128+32	T_0_ -24+2Ω	89+20	187+55
^T^3	23+61	117+9	154+39α	T_3_ -16+11	121+19	181+51
^T^6	26+45	120+17	144+30	T_6_ -14+11	113+11	150+15

Ω Behavior of Δ IV-EV at time T_0_ between the groups (*P*=0.0486).

α Variation of the HR from the instant T_0_ to T_3_ in the METT (*P*=0.0313).

METT=modified endotracheal tube; TETT=traditional endotracheal tube; T_0_=baseline; T_3_=3 hours after baseline; T_6_=6 hours after the baseline; Δ IV-EV=variation of the inhaled and exhaled volume; MAP=mean arterial pressure; HR=heart rate

Results expressed as mean ± standard deviation.

## DISCUSSION

Few studies have investigated pulmonary gas exchange with FiO_2_=0.21 during endotracheal intubation by using modified devices, both in clinical and in experimental practice.

Thus, besides being based on the relevant literature, the proposition of a distal cuff with variable pressure according to the respiratory cycle, aiming at high volume and low pressure, is not new. However, so far no ETT with modifications in the balloon proved to be didactical and practical.

The modification shown in the METT of this study consists in the insertion of three holes of 3 mm each in the distal cuff (Figure 1), which allows for inflation and deflation according to the IMV cycle^[4]^.

Statistical analysis regarding blood gas data confirmed the absence of hypoxia when both tubes were compared. This answered the question regarding the aforementioned device in terms of its effectiveness on the lung gas exchange, at least in a healthy body. It is relevant to point out that this study was carried out arbitrarily with an FiO_2_=0.21 in order to prove the real effectiveness of the device concerning hematosis.

Isolated analysis of a specific time (T_3_) in relation to HCO_3_ is not sufficient to attribute a likely ineffectiveness of the METT. This apparent occurrence of acidemia was normal in the subsequent time (T_6_), in which the same variable was not statistically significant. At T_3_, the same happened to the ΔIV-EV variable, which although statistically significant in relation to the average between the groups, by itselfit was insufficient to state that there was a possible problem in pulmonary gas exchange. Such an event could result in hypoxemia, which did not occur.

In addition, in relation to the pulmonary gas exchange, statistically significant values were found for IV and EV when T_0_ x T_3_, T_0_ x T_6_ and T_3_ x T_6_ were analyzed in pairs. Likewise, there were higher values of IV and EV in the METT than in TETT. However, that is not indicative of anything unexpected, since in the case of a balloon that inflates and deflates according to the respiratory cycle it is natural that the inspired and expired volumes will vary. A larger volume is needed in order to have a volume increase in the balloon, taking into consideration that a close volume (relatively small) is expelled at the time the device is emptied, which does not occur with the ETT, since it presents "constant" volume throughout the respiratory cycle.

If, on one hand, the aforementioned device was enough to prevent the installation of hypoxemia in this model, it should be noted that the effects arising from a possible sub-pressurization in the system could lead to the entrance of fluids in the intracuff region, a fact which was not proven by the Negro et al.^[3]^ study. Another attenuating element to the likely suction of secretions would be that the referred METT is specifically used in elective surgical procedures, particularly if the intubation time is short. However, further studies should be considered, especially in the case of pulmonary diseases.

**Table:** 

**Authors' roles & responsibilities**	
RQA	Study design; implementation of projects/experiments; analysis/interpretation of data; manuscript writing or critical review of its content; final approval of the manuscript
MMM	Study design; implementation of projects/experiments; analysis/ interpretation of data; manuscript writing or critical review of its content; final approval of the manuscript
LCM	Study design; implementation of projects and/or experiments; analysis and/or interpretation of data; manuscript writing or critical review of its content; final approval of the manuscript
MSDN	Manuscript writing or critical review of its content; final approval of the manuscript;
TAB	Study design; implementation of projects/experiments; manuscript writing or critical review of its content; final approval of the manuscript
AJT	Analysis and/or interpretation of data; study design; implementation of projects/experiments; manuscript writing or critical review of its content; final approval of the manuscript;

## Figures and Tables

**Table 2:** Blood gas data of the METT (n=7) and the TETT (n=3) Groups at T_3_.

T_3_												
	pH	PaO_2_	PaCO_2_	BE	hco_3_	Sat.O_2_	pH v	PvO_2_	PvCO_2_	BE v	HCO_3_ v	Sat.v O_2_
METT	7.52+0.02	92.4+6.36	39.9+1.4	8.7+1.5	31.3+1.6	96.0+1.6	7.46+0.02‡	44.1+6.7	48.7+2.0	9.0+1.8	32.9+1.9	58.6+5.7
TETT	7.46+0.02	94.3+7.9	39.3+1.0	4.2+0.8	26.7+0.6†	95.1+1.4	7.41+0.04	44.8+1.4	49.5+2.0	5.5+1.7	29.5+1.2	52.3+3.4

† Change in HCO_3_ at time T_3_ between groups (P=0.0486).

‡ pH variation between times T_0_ and T_3_ for MEDT (P=0.0469).

METT=modified endotracheal tube; TETT=traditional endotracheal tube; PaO_2_=partial pressure of oxygen in arterial blood; PaCO_2_=partial pressure of carbon dioxide in arterial blood; BE=base difference in arterial blood; HCO_3_=bicarbonate in arterial blood; Sat.O_2_=hemoglobin saturation in arterial blood; PvO_2_=partial pressure of oxygen in venous blood; PvCO_2_=partial pressure of carbon dioxide in venous blood; BE v=base difference in venous blood; HCO_3_ v=bicarbonate in venous blood; Sat.v O_2_=hemoglobin saturation in venous blood

Results expressed as mean ± standard deviation.

**Table 3:** Blood gas data of the METT (n=7) and the TETT (n=3) Groups at T_6_.

T_6_												
	pH	PaO_2_	PaCO_2_	BE	hco_3_	Sat.O_2_	pH v	PvO_2_	PvCO_2_	BE v	HCO_3_ v	Sat.v O_2_
METT	7.49+0.03	81.6+13.3	40.9+0.9	6.8+2.1	29.4+2.3	90.2+7.1	7.43+0.02	43.0+6.4	50.4+1.8	7.8+2.2	31.7+2.6	50.5+6.6¥
TETT	7.47+0.01	95.6+9.9	38.9+0.9	4.7+0.7	27.0+0.8	94.9+2.3	7.42+0.02	46.8+2.1	48.3+1.9	5.9+0.6	29.6+0.2	56.0+2.3

¥ Change of the Sat.v O_2_ between times T_3_ to T_6_ in MEDT (P=0.0156).

METT=modified endotracheal tube; TETT=traditional endotracheal tube; PaO_2_=partial pressure of oxygen in arterial blood; PaCO_2_=partial pressure of carbon dioxide in arterial blood; BE=base difference in arterial blood; HCO_3_=bicarbonate in arterial blood; Sat.O_2_=hemoglobin saturation in arterial blood; PvO_2_=partial pressure of oxygen in venous blood; PvCO_2_=partial pressure of carbon dioxide in venous blood; BE v=base difference in venous blood; HCO_3_ v=bicarbonate in venous blood; Sat.v O_2_=hemoglobin saturation in venous blood

Results expressed as mean ± standard deviation.
